# Liposomes for Intra-Articular Analgesic Drug Delivery in Orthopedics: State-of-Art and Future Perspectives. Insights from a Systematic Mini-Review of the Literature

**DOI:** 10.3390/medicina56090423

**Published:** 2020-08-20

**Authors:** Lucio Cipollaro, Paolo Trucillo, Nicola Luigi Bragazzi, Giovanna Della Porta, Ernesto Reverchon, Nicola Maffulli

**Affiliations:** 1 Department of Musculoskeletal Disorders, Faculty of Medicine and Surgery, University of Salerno, Via San Leonardo 1, 84131 Salerno, Italy; lcipollaro@unisa.it; 2 Department of Medicine, Surgery and Dentistry, University of Salerno, Via S. Allende, 84081 Baronissi (SA), Italy; gdellaporta@unisa.it; 3 Department of Industrial Engineering, University of Salerno, Via Giovanni Paolo II, 84084 Salerno, Italy; paolo.trucillo@unina.it (P.T.); ereverchon@unisa.it (E.R.); 4 Department of Chemical, Material and Industrial Production Engineering, University of Naples Federico II, Piazzale V. Tecchio, 80-80125 Napoli, Italy; 5 Laboratory for Industrial and Applied Mathematics (LIAM), Department of Mathematics and Statistics, York University, Toronto, ON M3J 1P3, Canada; bragazzi@yorku.ca; 6 Postgraduate School of Public Health, Department of Health Sciences (DISSAL), University of Genoa, 16132 Genoa, Italy; 7 Centre for Sports and Exercise Medicine, Barts and The London School of Medicine and Dentistry, Mile End Hospital, Queen Mary University of London, 275 Bancroft Road, London E1 4DG, UK; 8 School of Pharmacy and Bioengineering, Guy Hilton Research Centre, Keele University School of Medicine, Thornburrow Drive, Stoke on Trent ST4 7QB, UK

**Keywords:** liposomes, osteoarthritis, nanoparticles, vesicles, orthopedics, nanomedicine

## Abstract

*Background and objectives:* Liposomal structures are artificial vesicles composed of one or several lamellae of phospholipids which surround an inner aqueous core. Given the amphoteric nature of phospholipids, liposomes are promising systems for drug delivery. The present review provides an updated synthesis of the main techniques for the production of liposomes for orthopedic applications, focusing on the drawbacks of the conventional methods and on the advantages of high pressure techniques. *Materials and Methods:* Articles published in any language were systematically retrieved from two major electronic scholarly databases (PubMed/MEDLINE and Scopus) up to March 2020. Nine articles were retained based on the “Preferred Reporting Items for Systematic Reviews and Meta-Analyses” (PRISMA) guidelines. *Results:* Liposome vesicles decrease the rate of inflammatory reactions after local injections, and significantly enhance the clinical effectiveness of anti-inflammatory agents providing controlled drug release, reducing toxic side effects. *Conclusions:* This review presents an update on the improvement in musculoskeletal ailments using liposome treatment.

## 1. Introduction

Liposomal structures are artificial devices characterized by one or several lamellae of phospholipids which surround an inner aqueous core and form vesicles. Given the amphoteric nature of phospholipids, liposomes are promising systems for drug delivery [1]. 

Since their discovery by Dr. Alec Bangham over 40 years ago, liposomes have garnered considerable scholarly interest, and have been the topic of an extensive body of literature as drug delivery systems of bio-active molecules [2]. The original production of simple unilamellar vesicles resulted in low stability and easy early degradation immediately after exposure to heat. Second-generation liposomes were fabricated by coating the external lipidic surface using polyethylene glycol (PEG) [3]. PEG-based coating avoided degradation bio-processes, including phagocytosis induced by activation of the reticulo-endothelial system (RES), and, therefore, enabled to extend liposomes half-life. 

Moreover, the possibility of delivering loaded drugs to specific, selected target sites thanks to the creation of biochemical bonds among lipids and antibodies fragments or ligands contributed to raise the interest of the scientific community on liposomes [4] for their applications to human health and diseases [5]. 

The properties of liposomes are affected by a range of parameters, including surface charge, lipid composition, mean size, and the technique of formulation [6]. Hydrophilic compounds and lipophilic therapeutics can be incorporated within the inner aqueous compartment and in the lipidic double layer. The latter is optimal for drug delivery to the human body tissues and cells, given its similarity with biological components [7]. Various approaches and technologies can be used for the preparation, leading to liposomes ranging between 50 nm and 100 μm, based on the chosen production method[7], lipid composition, post-production step, filtration strategy, and number of lipid bilayers produced around water droplets, among others [7,8]. 

Most conventional techniques to fabricate liposomes are thin layer hydration method (or Bangham method), the extrusion method, microfluidic channel, and ethanol injection; they are largely used for several commercial application. 

For example, the microfluidic channel-based technique enables to produce homogeneous liposomes, given that the manufacturing of the channels size and shape of microsomes can be varied by varying flow rate and dilution in a microfluidic device. However, some authors described that some of these techniques have low replicability, low rate of encapsulation efficiency of the entrapped/loaded bio-compounds and high solvent residue in the final product [9]. For instance, in the microfluidic channel, the ethanol and the water phases are mixed together, and it is then quite difficult to remove the solvent from the solution.

For these reasons, several non-conventional, high pressure assisted processes have been devised to tackle these issues, including the “Depressurization of an Expanded Liquid Organic Solution” (DELOS), the “Supercritical Reverse Phase Evaporation” (scRPE), and the “Supercritical AntiSolvent” (SAS) production methods [10]. 

Despite these new technologies, there are still problems of high solvent residue, and encapsulation efficiencies are lower than 60% [11]. Therefore, a novel “supercritical assisted process” termed SuperLip (Supercritical assisted Liposome formation) has been recently designed and implemented to produce one-shot replicable vesicles at the nanometric level [12,13]. 

Liposomes have been largely employed in various fields, such as biomedical, pharmacological, and cosmetic ones. In particular, liposomes have been deployed to deliver chemotherapeutics to carcinogenic cells [14], cell signaling [15], vaccines to confer immunity [16], radiopharmaceuticals to improve and enhance diagnostic imaging [17], and gene therapy [18]. Several clinical trials have focused on liposomes, mostly for cancer and immunology applications [19,20]. 

This review will focus on the use of liposomes in the orthopedic field and in particular on a specific use as liposomes for “Intra-Articular Analgesic Drug Delivery”, as this is the only application actually present on the market. 

A general description of the technologies for liposome design and fabrication will be also discussed. focusing on the applications in the orthopedic field. However, molecules presently employed for the treatment of musculoskeletal illnesses are encapsulated into liposomes only using Bangham method, which causes massive drug loss resulting in increased costs. 

For this reason, a more successful process is needed to enhance drug loading for this application.

## 2. Materials and Methods

The article selection procedure was designed, carried out and reported adhering to the “Preferred Reporting Items for Systematic Reviews and Meta-Analyses” (PRISMA) guidelines [21]. 

A systematic search up to March 2020 was performed in PubMed/MEDLINE and Scopus scholarly databases evaluating the different applications of liposome formulations in the orthopedic field, with no language filter nor restriction in terms of the publication year. 

We utilized the terms “liposome”, “musculoskeletal”, “orthopaedic”, “orthopedic”, “orthopaedics” and “orthopedics” (as different possible spelling variants) variously combined as key terms. 

Articles designed as clinical case series or case reports, editorials, letters to the editor, brief reports, technical notes, commentaries, expert opinions, in vitro and animal studies, review articles (narrative and systematic) were excluded, even though the reference lists of the latter articles were scanned by hand to increase the chance to include all relevant articles. 

An orthopedic resident (LC) carried out the literature search and assessed the studies aided by a PhD student and chemical engineer (PT). Three independent researchers (NLB, GDP and NM) with expertise in the field of systematic reviews solved doubtful cases. Initially, the examiner read the title and abstracts of all the articles, and, based on pre-determined inclusion/exclusion criteria, selected the relevant ones, and then compared the results with the findings obtained by the other examiner. The extent of agreement was assessed by means of the kappa statistics. 

After initial familiarization, the same studies were read again two weeks later, to reach the consensus of the authors involved in the process of selection. No disagreements were observed among the investigators. Subsequently, the reviewers abstracted relevant information from the full-text articles to an *ad hoc* Excel structured spreadsheets to analyze each investigation. Possible discrepancies were discussed until they were solved. 

## 3. Results

### 3.1. Analysis of Results

**Figure 1:**
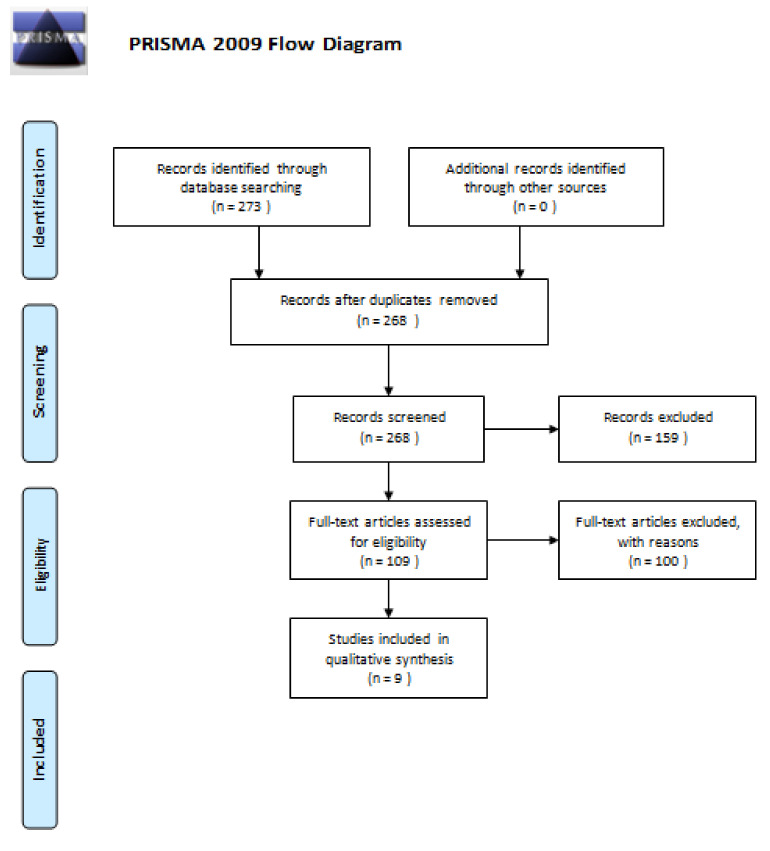
Preferred Reporting Items for Systematic Reviews and Meta-Analyses (PRISMA) flow diagram.

The initial literature search identified 273 records; with the exclusion of duplicates, 268 items were selected. The first inspection of the title and/or abstracts led to the exclusion of 159 articles. A further screening excluded 100 articles. A pool of 9 articles was retained and was selected for results synthesis and discussion (Figure 1).

The scholarly interest towards liposomes in the field of orthopedics has increased over time [22,23,24,25,26,27,28,29,30,31,32,33,34,35,36,37,38,39,40,41,42,43,44,45,46,47,48,49,50,51,52,53,54,55,56,57,58,59,60,61,62,63,64,65,66,67,68,69]. Table 1 summarizes the major characteristics of the studies included in the present systematic review.

### 3.2. Technologies for Liposome Design and Fabrication

Lipid nanocarriers can be used as smart nanomedicines able to provide site-specific targeting and delivery [22,23]. Nanomedicines are already present on the market [24], and represent a new frontier in modern therapeutics and clinical practice. This scenario is a solid evidence of the feasibility of suitable polymeric and/or lipid-based nanocarriers for the efficacious encapsulation and delivery of several drugs. The achievement of targeted drug delivery with high encapsulation efficiency and high cellular uptake of liposomes can be guaranteed only if the fabrication technique is solvent-free and has a 1-shot continuous configuration. 

In the last few decades, several conventional and non-conventional techniques have been proposed in the literature [25] (Table 2, Table 3 and Table 4). A large number of clinical trials have used liposome formulations, mostly for cancer treatment (see Table 4). Most of these formulations have been also commercialized, after approval by the Food & Drug Administration (FDA) [19]. 

Recently, Supercritical assisted Liposome formation (SuperLip) technology-based approach using different compositions has been proposed to produce liposomes at namometric level, with potential applications in various industrial fields, such as the pharmacological, cosmetic and nutraceutical ones [15,38]. This technology allowed to fabricate Single Unilamellar Vesicles (SUV) optimizing particle size and distributions (both at nanometric and micrometric level) with high encapsulation efficiency in both lipid and water phase [39]. 

To favor ethanol extraction from liposome suspension, SuperLip uses dCO_2_ to improve lipid/ethanol/water mixing. Recently, phosphatidyl-choline (PC) small unilamellar vesicles with an average size of 0.2 ± 0.05 μm of were loaded with Fluorescein Iso-ThioCyanate (FITC), using a lipid concentration of at 8 μg/mgPC. Liposomes loaded bioavailability was monitored by incubation with human monocytes isolated from the blood of healthy donors’ by flow cytometer assay, which represents the only cell population that could properly internalize the carriers. An internalization of 96.1 ± 21% was obtained, at a dosage of 0.1 mg/mL for SuperLip fabricated nanocarriers, with a monocytes viability of almost 100% at all the concentrations studied after vesicles internalization. This result suggested the reliability of the dCO2 technologies, opening perspectives for future drug loading [40]. 

### 3.3. Liposomes for Intra-Articular (IA) Injections

The major obstacle for drug transport out of the joint space is represented by the synovium. In the joint cavity, molecules of soluble drugs released from the immobilized depot undergo various distribution processes and reactions [41]. For the transport of small molecules, the extra-cellular matrix is the main diffusional barrier, whereas the endothelium represents the major barrier for the diffusion and transport of proteins [41]. Therefore, the drug formulation sizes and their passage through the articulation determine their reliability for cellular uptake and tissue penetration. 

That is why the dimension of the formulation is a key point in intra-articular (IA) drug delivery. Hence, IA injection of active molecules could be ineffective without the use of a drug carrier, since small molecules are rapidly cleared from these tissues. Native drugs are cleared from the joint space just in a couple of hours through lymphatic drainage [42]. For instance, the half-lives of methotrexate, ibuprofen and diclofenac are 0.59–2.9, 1.9 and 5.2 h, respectively [41]. IA drug delivery systems are expected to solve the issue of the low persistence times because of the quick uptake of the drugs injected within the synovial cavity, which determines adverse side effects and low bioavailability. Considering their structure, liposomes provide controlled drug release [43]. There are still no studies on human patients that demonstrate the efficacy of IA liposome treatment, but several studies on animal models have produced encouraging results [42,44,45,46,47]. Liposome vesicles reduce the incidence of inflammatory reactions after local injections compared to crystalline drug suspensions [48]. 

Furthermore, the IA delivery of several non steroidal anti-inflammatory drugs could avoid the risk of gastric side effects and cardiovascular problems intrinsic with their systemic administration [10].

### 3.4. Liposomes in Postsurgical Analgesia

Orthopedic surgery is frequently associated to remarkable postoperative pain [49], which may continue for 2 years or even longer [50]. About 50% of the patients who undergo joint arthroplasty experience intense postsurgical pain [51]. Inappropriate postsurgical pain management may cause development of chronic pain, thromboembolic or pulmonary complications, and decrease in health-related quality of life [52]. In orthopedic surgery patients, the inability to effectively control postsurgical pain has been associated with reduced capacity for exercise, delayed time to ambulation, and increased hospital length of stay [53,54]. A prolonged-release injectable liposomal formulation of bupivacaine, a local anesthetic, is available (Exparel®; Pacira Pharmaceuticals, Inc., Parsippany, NJ, USA) and can be injected at the surgical site to produce postsurgical analgesia [55,56]. 

The mechanism of action of Exparel is similar to that of marcaine and other local anesthetics, but its pharmaco-kinetic profile is unique [55]. With multiple aqueous chambers, Exparel is a multivesicular formulation enabling prolonged release and rapid absorption of bupivacaine when injected locally. To produce long-lasting effects, Exparel has a bimodal pharmacokinetic profile: after administration at the surgical site, bupivacaine diffuses slowly out of the chambers, with an initial peak in plasma concentration within the first hour after injection, and a second peak 12 to 36 h later [55]. Compared to placebo and bupivacaine hydrochloride (HCL), a single administration of liposome bupivacaine provided postsurgical analgesia for up to 72 h, reduced postsurgical opioid consumption, and delayed the use of rescue medication [57]. Liposome bupivacaine did not reduce postoperative pain when compared to other local anesthetics at 24 or 48 h after surgery [58], and did not reduce postoperative opioid uptake at different time-points (namely, at 24, 48, and 72 h) [59,60,61]. Liposome bupivacaine does not exhibit an analgesic advantage when compared to plain local anesthetics for patients undergoing surgical procedures. Premkumar et al. [62] evaluated whether the use of liposomal bupivacaine after anterior cruciate ligament (ACL) reconstruction would decrease opioid use and pain when compared with the same volume of 0.25% bupivacaine HCl. There were no significant differences in postoperative opioid use and postsurgical pain comparing patients receiving liposomal bupivacaine with those receiving 0.25% bupivacaine HCl [62]. Schroer et al [63], instead of standard bupivacaine in periarticular injections (PAI), used liposomal bupivacaine as part of a multimodal pain management, and did not evidence significant benefit after primary total knee arthroplasty (TKA). No significant differences in pain scores were found, as well as no differences in narcotic use during hospitalization, with no variations in hospital length of stay. Similar findings have been reported by Bagsby et al. [64]: in 150 patients, they found less pain relief in periarticular injection of liposomal bupivacaine compared to a combination of ropivacaine, epinephrine and morphine. The authors explained the unsatisfactory outcomes of the liposomal bupivacaine by the slow release of the drug from the liposomal structures, limiting the availability of free bupivacaine at the site of action [63,64]. 

Webb et al. [65], differently, found that after a TKA, healthier patients and those with a BMI <40 had a shorter hospital stay and used fewer narcotics with the use of liposomal bupivacaine. The same results were achieved by Mont et al [66], who in 140 patients showed reduced postsurgical pain, and decreased opioid uptake, and increased time to first opioid rescue after a TKA. Bramlett et al. [61] investigated 138 TKA patients comparing the effectiveness, safety profile, and pharmacokinetics of liposome bupivacaine with 150 mg of bupivacaine hydrochloride. Liposome bupivacaine was associated with statistically significantly greater analgesia while patients were at rest after surgery compared with bupivacaine hydrochloride. Alter et al. [55] compared the effect of Marcaine and Exparel in patients with distal radius fractures. Patients who received Exparel experienced less pain on the day of surgery but no difference in the following 5 days; they also consumed fewer opioids on the day of surgery, with no difference in the following days. An interesting effect noted comparing local anesthetics with liposome bupivacaine was the reduction of postoperative nausea [67]. The mechanism responsible for the antiemetic effect of liposome bupivacaine remains to be determined, but the reduction of postoperative nausea is an important goal in peri-operative patients [68]. In the largest case-control study to date, Barrington et al. [69] performed more than 2000 hip and knee joint arthroplasties adopting a standard multimodal pain care protocol with a periarticular injection, or a protocol including a periarticular injection of liposomal bupivacaine. In patients managed with liposomal bupivacaine, visual analog scale pain scores were found to be lower in a statistically significant fashion for both hip (1.67 versus 2.30; *p* < 0.0001) and knee (2.21 versus 2.52; *p* < 0.0001) procedures. Furthermore, the number of pain-free patients increased and the overall costs decreased [69].

### 3.5. Liposomes Can Help Prevent Orthopedic Device-Associated Osteomyelitis

Osteomyelitis, caused by bacteria contamination at the time of surgery, systemic transmission, direct colonization, or orthopedic device implantation, remains a major challenge for orthopedic surgeons [70]. Post-arthroplasty infection still occurs in 1.2% of primary arthroplasties and 3–5% of revisions, despite antibiotics being commonly used for prophylaxis [71]. These complications often result in significantly worse patients outcomes. 

Liu et al. [72] tried to devise a technique to counteract osteomyelitis associated with orthopedic arthroplasty. They successfully developed a novel alendronate-based binding liposome formulation to prevent orthopedic implant associated osteomyelitis. The alendronate-based binding portion was conjugated to cholesterol, demonstrating fast and strong binding capability to a model implant surface. The biomineral-binding liposome formulation added with oxacillin reliably prevented bacterial colonization compared to controls when challenged with a *Staphylococcus aureus* isolate [72].

### 3.6. Liposomes in Hirudo Therapy for OA

Non steroidal anti-inflammatory drugs are commonly used to relieve pain associated with osteoarthritis (OA). However, the incidence of adverse effects is high. Thus, researchers have attempted to use organic products, such as leech saliva, to achieve safe and alternative painkillers. The process of blood-letting and purification, in medicine practices like Leech therapy (LT) or Hirudotherapy, relieves a variety of chronic diseases such as blood disorders, gout, and skin disorders [73,74]. Introduced by the FDA, leech is a modern therapeutic agent and it contains different peptides and proteins such as histamine, steroid hormones and modulators, serotonin, enzymes, anti-microbial agent, and protease inhibitors. These substances exhibit analgesic, anti-inflammatory, thrombolytic, vasodilation, and anticoagulation effects, which improve blood circulation and relieve several ailments [75]. Leech saliva may block the cascade involved in certain steps of the modulation and regulation of pain via cytokines hindering from the anti-inflammatory agents present in the saliva [76]. 

Shakouri et al. [77] extracted the saliva of medical leech, and a nano liposomes-based gel was used to formulate the supplement to enhance skin absorption. Pain was relieved up to 50% after one month of administration of leech saliva liposomal gel. Also, given the reduction in stiffness and joint inflammation, the patients quality of life was enhanced (*p* < 0.001) and the range of motion was increased.

## 4. Discussion

Orthopedic surgery procedure can induce severe post-operative pain [78]. The general principle, common to all disciplines (not only the surgical ones), at the basis of the prevention and management of postoperative pain is represented by the combination of multiple techniques and therapeutic agents, which act at every level of the pain conduction pathways [79,80,81,82]. Enhancing the effect of these analgesic techniques and reducing the dose of drugs to be administered, consequently reducing adverse effects, are the main objectives of research in this area. 

In this context, the development of long-lasting anesthetic or pain-relieving formulations are increasingly being used in clinical practice [83,84,85]. Liposomal formulations, which allow the encapsulation of pharmaceutical agents, prolong the residence time at their site of action [58]. However, the scholarly literature on the therapeutic superiority of liposomal formulations compared to standard analgesic formulations is conflicting [58]. Further studies should focus on what the optimal drug dose should be, in relation to the different types of surgery and the potential adverse effects of the drug. In terms of clinical practice and implications, the use of liposomal formulations is not yet widespread, and therefore developing research and studies on these formulations would be of paramount importance.

Liposomes are potentially highly efficient drug delivery systems, especially for biomedical and orthopedic applications. Liposomes exhibit high cell penetration and efficacy, particularly if they are produced at the nanometric level, using novel techniques and approaches such as the SuperLip technology. Liposomes loaded with molecules for OA treatment showed an enhanced half-life and provided controlled drug release, reducing toxic side effects. The major benefit of these formulations is the possibility to provide a delayed and controlled drug release, thus resulting in a substantial reduction in the number of administration procedures. Indeed, the main reason why liposomal formulation-based bupivacaine has not been widely adopted is its cost. At US$ 283.00 per 20 mL, it is significantly more expensive than 20 mL of 0.5% bupivacaine HCl: a 30 mL vial costs, indeed, US$ 1.24. For this reason, the higher costs of liposomal products should be compensated by greater efforts to reduce manufacturing cost. This is not particularly simple, since it depends also on the drug synthesis, production line, purification and quality control post-processing steps. 

The current literature evidenced improved outcomes administrating liposomal formulations. Further statistically robust evidence of reasonable costs is warranted to draw solid and robust conclusions concerning the benefits of this approach. The relationship between cost and benefits not only arises from the price of the medication but also from other factors such as the use of pain pumps and opioids, operative time, hospitalization time, and readmissions. Even though the cost of liposomal bupivacaine is clearly higher than that of bupivacaine hydrochloride, information extrapolated from a retrospective study showed a clear reduction in hospitalization costs compared with standard care [78,79]. Very interesting indications are provided by a case-control study of more than 2000 joint arthroplasties conducted adopting a classical multimodal pain care protocol with periarticular injection versus targeted delivery of liposomal bupivacaine. This study showed a significant improvement in pain outcomes and a mean decrease in hospital overall direct cost of US$1,246 per patient using liposomal bupivacaine [69]. 

For this reason, liposomes appear promising drug delivery systems for orthopedic applications.

## 5. Conclusions

Liposomes are potentially highly efficient drug delivery systems, especially if they are produced at the nanometric level, using advanced and sophisticated techniques and approaches such as the SuperLip technology. Liposomes loaded with molecules for osteoarthritis (OA) treatment showed an enhanced half-life and provided controlled drug release, reducing toxic side effects and exhibiting cost-effectiveness. As such, liposomes could be powerful and cheaper drug delivery systems for orthopedic and, in general, biomedical application.

## Figures and Tables

**Table 1:** Summary of the studies included in this review.

Authors (Year)	Study Design	Sample Size	Age	Gender	Disorder	Procedures	Treatment	Result	Adverse Outcomes
Alter et al. (2017) [55]	Prospective, randomized, single-blinded, single-center clinical trial, sample size *a priori* computed, systematic recruitment	41, 20 receiving Exparel, 21 receiving marcaine	63 ± 15 years receiving Exparel, 57 ± 15 years receiving marcaine	16 women (80%) receiving Exparel, 17 (81%) receiving marcaine	Distal Radius Fracture	Distal Radius Fracture Repair Surgery	Exparel 20 mL+ 10 mL 0.5% Marcaine	Exparel use resulted in decreased pain (4.0 versus 6.0, *p* < 0.05) and opioid consumption (1.2 versus 2.0 pills, 7.3 versus 12.5 oral morphine equivalents) only on the day of surgery and not thereafter	16/20 receiving Exparel and 11/21 receiving marcaine experienced hand numbness, 1/20 receiving Exparel and 4/21 receiving marcaine reported itching, nausea, drowsiness/dizziness, and lack of energy
Amundson et al. (2017) [60]	Three-arm, parallel, single blinded (outcome adjudicator-blinded), superiority, randomized-controlled, single-center clinical trial, sample size *a priori* computed, systematic recruitment	157 (out of an initial list of 165 patients), 52 receiving Exparel, 55 receiving Ropivacaine, 50 receiving peripheral nerve block	67 ± 8 years receiving Exparel, 68 ± 8 years receiving Ropivacaine, 67 ± 9 years receiving peripheral nerve block	27 women (52%) receiving Exparel, 34 (62% receiving Ropivacaine), 25 (50%) receiving peripheral nerve block	Patients needing total knee arthroplasty	Elective, Unilateral, Primary, Total Knee Arthroplasty	Exparel 20 mL (266 mg) + 100 mL Saline +120 mL (300 mg) Ropivacaine	No significant benefit of liposomal bupivacaine over ropivacaine in periarticular injections for total knee arthroplasty (post-operative day 1 median maximal pain score was lower for peripheral nerve blockade, *p* = 0.016, median difference -1 [95%CI -2 to 0]), patients receiving Exparel exhibited improved physical quality of life (*p* = 0.048), as well as those receiving Ropivacaine (*p* = 0.001), but not those receiving peripheral nerve block	6 patients fell (2 receiving peripheral nerve block, 1 receiving Ropivacaine, 1 receiving Exparel), 6 patients had a wound infection (2 for each group)
Bramlett et al. (2012) [61]	Phase 2, randomized, parallel-group, double-blinded, dose-ranging, multi-center clinical trial, sample size *a priori* computed, systematic recruitment	138 (out of an initial list of 164 screened patients and of 144 randomized patients), four discontinued the trial, two experienced serious adverse events, one died, one left for other reasons, 27 receiving DepoFoam 133 mg, 25 receiving DepoFoam 266 mg, 26 receiving DepoFoam 399 mg, 24 receiving DepoFoam 532 mg, 32 receiving Bupivacaine	61.4 ± 7.0 years receiving DepoFoam 133 mg; 61.1 ± 8.7 years receiving DepoFoam 266 mg; 61.8 ± 6.3 years receiving DepoFoam 399 mg, 64.9 ± 7.3 years receiving DepoFoam 532 mg, 62.2 ± 7.2 years receiving Bupivacaine 150 mg	15 women (53.6%) receiving DepoFoam 133 mg; 12 women (48.0%) receiving DepoFoam 266 mg; 15 women (57.7%) receiving DepoFoam 399 mg; 20 women (80.0%) receiving DepoFoam 532 mg; 23 women (67.6%) receiving Bupivacaine 150 mg	Patients needing total knee arthroplasty	Unilateral, Primary, Total Knee Arthroplasty	Exparel 20 mL (266 mg) + 40 mL Saline + 50% Bupivacaine 30 mL + 30 mL NS	Exparel was associated with statistically significantly greater analgesia compared with bupivacaine HCl in terms of pain at rest and pain with activity	Overall 112 (81.2%) experienced at least one side-effect (79.8% receiving DepoFoam versus 85.3% receiving Bupivacaine)
Premkumar et al. (2016) [62]	Prospective, double-blinded, randomized, positive-controlled, single-center clinical trial, systematic recruitment	32 (out of an initial list of 35 patients), follow-up rate of 90.6%, 16 receiving Exparel, 16 receiving Bupivacaine	24.1 ± 7.3 years receiving Exparel, 25.5 ± 6.8 years receiving Bupivacaine	33% women receiving Exparel, 47% women receiving Bupivacaine	Injury of the anterior cruciate ligament	Anterior Cruciate Ligament Reconstruction with a soft tissue quadriceps tendon autograft	Exparel/Bupivacaine 20 mL + 20 mL 0.9% Saline	No significant differences in postoperative pain, recovery time, mobility, pain location or opioid use between patients receiving liposomal bupivacaine or 0.25% bupivacaine HCl	Not reported
Schroer et al. (2015) [63]	Prospective, randomized, clinical trial, systematic (consecutive) recruitment	111, 58 receiving Exparel, 53 receving Bupivacaine	67 ± 8.8 (48–86) years receiving Exparel, 68.6 ± 9.2 (52-89) receiving Bupivacaine	34 women (59%) receiving Exparel, 32 women (60%) receiving Bupivacaine	Patients undergoing a total knee arthroplasty	Unilateral, Cemented Total Knee Arthroplasty through a mini-subvastus approach, anteriorly stabilized, with resurfacing of patelle	Exparel 20 mL (266 mg) + 30 mL 0.25% Bupivacaine + 0.25% Bupivacaine 60 mL	Liposomal bupivacaine did not demonstrate improved pain scores, lower narcotic use, or better knee motion during hospitalization	3 cases (5%) and 2 controls (4%) had post-operatve nausea
Bagsby et al. (2014) [64]	Retrospective, cohort study, systematic (consecutive) recruitment	150; 65 receiving Exparel, 85 receiving Ropivacaine	63.13 ± 10.32 years receiving Exparel, 65.19 ± 9.21 years receiving Ropivacaine	47 (72.3%) women receiving Exparel, 61 (70.9%) women receiving Ropivacaine	Patients undergoing total knee arthroplasty	Total Knee Arthroplasty	Exparel 20 cc + 30 cc Saline + 30 cc 0.5% Marcaine	Exparel provided inferior pain control compared to Ropivacaine (*p* = 0.04), being more expensive	In the Exparel group, 3/65 patients (4.6%) reported a wound drainage at 3–4 weeks post-surgery and an acute postoperative methicillin sensitive staphilococcal infection requiring reoperation
Webb et al. (2015) [65]	Retrospective, case-control study, systematic (consecutive) recruitment	100; 50 receiving Exparel, 50 serving as controls	64 (46–88) years receiving Exparel, 64 (38–85) years serving as controls	34 (68%) women receiving Exparel, 32 (64%) serving as controls	Patients undergoing total knee arthroplasty	Total Knee Arthroplasty	Exparel 20 mL (266 mg) + 40 mL Saline	Use of Exparel resulted in decreased narcotic usage (60.97 mg oral morphine equivalent versus 89.74 mg, *p* = 0.009). Periarticular Total Knee Arthroplasty injection using liposomal bupivacaine in patients with a Body Mass Index less than 40 kg/m^2^ and few co-morbidities lead to earlier hospital discharge ((2.64 days versus 3.06 days, *p* = 0.004) and decreased narcotic usage over 24–48 h (110.66 mg versus 182.47 mg, *p* = 0.013), and over 48–72 h (49.61 mg versus 112.65 mg, *p* = 0.004)	Not reported
Mont et al. (2018) [66]	Phase 4, randomized, double-blinded, active-controlled, parallel-group, multi-center clinical trial, sample size *a priori* computed, systematic recruitment	139 (out of an initial list of 140 patients), 70 receiving Exparel, 69 receiving Bupivacaine	66 ± 8.61 years receiving Exparel, 66 ± 7.21 years receiving Bupivacaine	43 women (61.4%) receiving Exparel, 39 women (56.5%) receiving Bupivacaine	Patients with degenerative knee osteoarthritis undergoing total knee arthroplasty	Primary, Unilateral, Tricompartimental, Total Knee Arthroplasty	Exparel 20 mL (266 mg) + 40 mL Saline + 50% Bupivacaine 20 mL	Exparel provides significantly reduced postsurgical pain (area under the curve of visual analog scale pain intensity score 12–48 h post-surgery 180.8 versus 209.3, *p* = 0.0381), reduced opioid consumption (18.7 mg versus 84.9, *p* = 0.0048), percentage of patients (*p* < 0.01), and time to first opioid rescue (*p* = 0.0230)	64.3% receiving Exparel versus 56.5% receiving Bupivacaine experienced mild-to-modest adverse events
Barrington et al. (2015) [69]	Prospective, randomized clinical trial, sample size power calculated *a posteriori*, systematic (consecutive) recruitment	2248; 1124 receiving a classical, well-established multimodal analgesia, including peri-articular injection, 1124 receiving Exparel (pre-post design)	63.1 (19.0–95.0) years receiving the multimodal analgesia, 65.8 (32.0–96.0) years receiving Exparel, for hip procedures, 66.7 (36.0–93.0) years receiving the multimodal analgesia, 66.7 (38.0–97.0) years for receiving Exparel, for knee procedures	56.5% women receiving the multimodal analgesia, 57.2% receiving Exparel, for hip procedures, 58.7% women receiving the multimodal analgesia, 57.5% receiving Exparel, for knee procedures	Patients undergoing knee/hip arthroplasty	Knee/Hip Arthroplasty (primary knee, 48%, revision knee, 45%, unicompartmental knee, 56%, bilateral knee, 46%, primary hip, 50%, revision hip, 47%, and bilateral hip, 50%)	Exparel versus multimodal analgesia	Improved overall mean VAS pain scores for hip (1.67 versus 2.30, *p* < 0.0001) and for knee (2.21 versus 2.52, *p* < 0.0001) procedures, an increased number of pain-free patients, decreased hospital length of stay (*p* < 0.0001), trends toward decreased falls (*p* = 0.021), and decreased overall cost	Not reported

**Table 2:** List of techniques for liposomes fabrication and their disadvantages.

Techniques	Disadvantages	Author (Year)
Bangham method	(1) Large particle size distribution, that means production of large vesicles (mean size > 10 µm), that are not compatible with pharmaceutical applications (2) Low replicability, i.e., production of heterogeneous vesicles, that are not applicable to industrial production (3) High solvent residue, i.e., high toxicity and low biocompatibility to human tissues (4) Low encapsulation efficiency (<30%), i.e., low loading efficacy of drugs, resulting in a high percentage of drug waste	Bangham et al. (1974) [26]
Extrusion method		Mui et al. (2003) [27]
Microfluidic channel		Andar et al. (2014) [28]
Ethanol Injection		Charcosset et al. (2015) [29]

**Table 3:** List of non-conventional techniques for liposomes’ fabrication.

Techniques	Disadvantages	Author (Year)
Supercritical reverse phase evaporation	(1) Semi-continuous processes, meaning that the process cannot be replicated at large scale, for example for the massive fabrication of liposomes for vaccine delivery (2) Encapsulation Efficiency of drugs <60%. Higher than conventional methods, but still too low to obtain a large profitability from the process (3) Low stability, i.e., vesicles are not stable over a long observation time (4) Difficult control of particle size distribution linked to problems of replicability	Otake et al. (2006) [30]
Depressurization of an Expanded Solution into Aqueous Media		Meure et al. (2009) [31]
Depressurization of an Expanded Liquid Organic Solution		Zhao, Tamelli (2015) [32]
Supercritical Anti-Solvent		Lesoin et al. (2011) [33]

**Table 4:** List of the most commercialized liposome formulations.

Commercialized Liposomes Formulation	Commercial Name	Author (Year)
PEGylated liposomal doxorubicin	(Doxil/Caelyx)	Gabizon et al. (2003) [34]
Non-PEGylated liposomal doxorubicin	(Myocet)	Rivankar (2014) [35]
Liposomal daunorubicin	(DaunoXome)	Petre, Dittmer (2007) [36]
Liposomal cytarabine	(DepoCyt)	Bomgaars et al. (2004) [37]

## Abbreviations

RES: Reticulo-Endothelial System, PEG: polyethylene glycol, scRPE: Supercritical Reverse Phase Evaporation, SAS: Supercritical Anti-Solvent, DELOS: Depressurization of an Expanded Liquid Organic Solution, DESAM: Depressurization of an Expanded Solution into Aqueous Media, SuperLip: Supercritical assisted Liposome formation, PRISMA: Preferred Reporting Items for Systematic Reviews and Meta-Analyses, FDA: Food and Drug Administration, SUV: Single Unilamellar Vesicles; FITC: Fluorescein Iso-ThioCyanate; IA: Intra-Articular; PC: Phosphatidyl-Choline.
